# The Psychological Distance of Modern Slavery Risk

**DOI:** 10.1111/risa.70110

**Published:** 2025-10-12

**Authors:** M. Selim Cakir, Jamie K. Wardman, Alexander Trautrims

**Affiliations:** ^1^ Birmingham Business School University of Birmingham Birmingham UK; ^2^ University of Leicester School of Business, University of Leicester Leicester UK; ^3^ Nottingham University Business School, University of Nottingham Nottingham UK

**Keywords:** construal‐level theory, ethical organizational climate, forced labor, modern slavery, psychological distance

## Abstract

Modern slavery has become recognized as one of the world's great human rights challenges due to the high prevalence of coercive labor exploitation associated with the production and consumption of many goods and services across the globe. Yet, while its practice is commonly considered to be “unseen” and far removed from many people's everyday lives and working experiences, the micro‐level bases of individual perceptions and actions taken in response to this “distal” threat remain poorly understood. In this paper, we develop and test a model linking the “psychological distance of modern slavery risk” to individual concerns, ethical organizational climate, and intentions to engage in mitigating behaviors in the workplace. Results from a survey of 511 working adults from UK businesses show that “closer” psychological distance to modern slavery is associated with higher levels of concern and greater intention to act in response to this risk. We also find that ethical climate moderates the impact of modern slavery risk concerns on intentions to engage in mitigating behaviors. Our study findings, therefore, complement existing research by pinpointing the key roles of psychological distance and ethical climate in modern slavery risk responses and highlighting the potential for micro‐level interventions to help promote antislavery action.

## 1 Introduction

Modern slavery has been utilized as an umbrella term for the worst forms of coercive exploitation affecting cases of forced labor, human trafficking, and domestic servitude found to be prevalent across the world today (Bales 1999; Crane et al. 2022; Le Baron 2021; Tickler et al. 2018). The latest figures produced by the Global Slavery Index estimate the number of people in modern slavery around the world to be approaching 50 million, with approximately 17.3 million thought to be in private sector forced labor (International Labour Organization 2022). The heightened prevalence of modern slavery has been accompanied by growing research interest into its possible causes and solutions (Andersson et al. 2019; Caruana et al. 2021). At a macro‐level, studies indicate that issues such as the spread of global capitalism alongside weak regulation and governance are commonly associated with the higher prevalence of modern slavery across many different nations and business sectors (Landman and Silverman 2019; Le Baron 2021; Tickler et al. 2018). At the organizational level, further studies have shown that factors such as irresponsible business strategies and procurement models contribute to the heightened risk that goods and services are tarnished by modern slavery (Gold et al. 2015).

However, alongside these developments, some exploratory research at the micro‐level suggests that for many businesses and consumers, modern slavery is experienced as a “distal” threat that is not typically regarded as a meaningful or relevant day‐to‐day concern, especially for those who reside and work in nations with advanced economies (Andersson et al. 2019; Carrington, Chatzidakis, and Shaw 2021; Simpson et al. 2021). This elision may stem in part from a general lack of awareness, but it can often be difficult for individuals to clearly identify possible instances of modern slavery because its warning signs can be ambiguous and hard to interpret (Crane et al. 2022). This ambiguity may also be exacerbated by the problem that legal frameworks often rely on binary categorizations of modern slavery, which classify individuals as either “enslaved” or “free,” and in doing so fail to adequately account for the full spectrum of worker vulnerability to labor restrictions and exploitation (Crane et al. 2022). Understanding the role and influence of individual appraisals and outlooks towards the distal threat posed by modern slavery is therefore a priority because the micro‐level components of decision processes are often found to be crucial to what actions are taken against risk in the absence of wider reform (Chou et al. 2008; Waller et al. 2004). In the case of modern slavery, individuals can take practical measures, such as considering this risk in purchasing decisions, reporting suspected cases to authorities, or discussing the issue with colleagues to raise awareness and prompt changes to other's behavior as well as their own.

One of the few exploratory studies investigating experiential understandings and perceptions of modern slavery risk found that, despite a general acknowledgement that modern slavery was ethically wrong, levels of concern towards workers remained low (Stringer et al. 2022). Nevertheless, a second qualitative study addressing perceptions of modern slavery within a work context further found that variable attention to different qualitative features of the problem could influence considerations of what subsequent actions ought to be undertaken in response (Simpson et al. 2021). Yet, to date, besides a small number of exploratory qualitative studies, there remains a notable lacuna in empirical evidence supporting the role of micro‐level processes in shaping modern slavery risk appraisals both generally and within the workplace. Subsequently, important questions remain concerning how the distal features of modern slavery risk are commonly perceived, what association this might have with levels of concern and mitigating behaviors, along with how this might interact with wider organizational norms and values in the workplace.

To help address these research gaps, this study investigated the “psychological distance” of modern slavery risk through a survey of 511 employees from UK companies, which examined how the distal qualities of this risk impact people's perceptions, concerns, and behavioral intentions to mitigate the problem within their workplace. As modern slavery often operates in the absence of strong regulatory oversight and enforcement, but appraisals of this risk nevertheless retain a strong moral component (Carrington et al. 2021; Stringer et al. 2022), the study also investigates the role and influence of “ethical climate” (David et al. 2021; Victor and Cullen 1987; Wang and Hsieh 2013) within organizations on these associations.

## 2 Theoretical Background

### 2.1 Construal‐Level Theory and Psychological Distance

According to Trope and Liberman (2010), people perceive phenomena at different levels of abstraction depending on the representations they can directly draw upon and form. For instance, when phenomena are construed at high levels of abstraction, individuals will attend to their broader, inclusive, and global features, whereas construal at a low level of abstraction will reflect attention to peripheral, subordinate, and local features (Trope and Liberman 2010). Following construal‐level theory, variability in the level of construal employed is argued to affect the way that individuals consider objects and events in different ways, such as how they might appraise the desirability and feasibility of preferred options and outcomes, along with the subsequent behavior that they enact (Bornemann and Homburg 2011). Importantly, perceptions and behaviors can also be modified by individuals as corresponding levels of abstraction and construal change, such as when new information is presented or by obtaining more direct personal experience of an issue that introduces other criteria for consideration (Kirshner and Moritz 2023).

In accordance with construal‐level theory, the concept of “psychological distance” has been introduced to denote the extent to which individual conceptualizations are perceptually “removed” from a target object, activity, or event (Trope and Liberman 2010). Specifically, psychologically “distant” events are construed through attentiveness to “high‐level” abstract features, whereas psychologically “close” events are construed according to “low‐level” concrete features (Chu and Yang 2018; Wiesenfeld et al. 2017). Psychological distance is also considered to contain different fundamental dimensions, which include hypotheticality (the likeliness of a phenomenon), temporality (how soon something will occur), social similarity (if people who are similar or different to the person concerned are affected), and spatiality (whether something is geographically close or far away) (Spence et al. 2012). When individuals construe events as being psychologically closer, they are perceived as probably occurring, happening soon, affecting similar people, and taking place nearby (Trope and Liberman 2010). Conversely, when phenomena are construed by individuals as more psychologically distant, they are seen as less probable, to occur later, affect people who are dissimilar, and happen far away (Liberman and Trope 2008; Trope and Liberman 2010). This theory suggests that changes in psychological distance can accordingly alter the criteria that people consider when making decisions, such as whether to act or not in response to an issue like climate change being distant or far (Spence et al. 2012).

The influence of psychological distance has subsequently been found across a wide range of domains, including organizational politics (Maslyn et al. 2017), climate action (Kim 2023, 2024; Spence et al. 2012), entrepreneurship (Chen et al. 2018), leadership (Cole et al. 2009), consumer preferences (Bornemann and Homburg 2011), the evaluation of products (Jia et al. 2021), and inventory decision‐making (Kirshner and Moritz 2023). This latter work in particular underscores the concern that the spatial and temporal distances which are commonly a natural feature of supply chains can lead to cognitive biases in decision‐making (Kirshner and Moritz 2023). To date, there has been limited application of the concept of psychological distance to examine people's experiential understanding and appraisals of the risk of modern slavery. Nevertheless, there are a number of reasons and some evidence to suggest that it may be useful not only to understand people's appraisals of modern slavery risk but also that reducing psychological distance may be crucial to altering the criteria that people consider when deciding how best to address the problem (Simpson et al. 2021).

### 2.2 The Distal Qualities of Modern Slavery

Although the precise meaning of *modern slavery* can be interpreted in different ways, studies on the topic have produced some instructive insights and analysis that have generated some small areas of agreement about the scope and scale of the problem, along with how it might be resolved. For instance, research typically acknowledges that whereas most victims of modern slavery typically reside in developing countries, no industry can be said to be free of this risk (Christ et al. 2020). Rich nations are also widely understood to be the primary beneficiaries of goods and services derived from forced labor, and dependence on its practice has seemingly become tacitly embedded within the business models of many companies, particularly multinational corporations (Shilling et al. 2021).

Following recognition of the scope and scale of this variegated problem, modern slavery has accordingly been identified as one of the world's greatest human rights challenges and included in the United Nations Sustainable Development Goals (UNSDG 8.7). A range of national and international regulatory instruments and agreements have also been introduced, albeit with different interpretations of the problem of modern slavery and what to do about it. For instance, whereas the US approach is primarily centered around an “import ban” for products made using slave labor, France and Germany have focused on implementing “due diligence” laws that require large businesses to act against the risk of modern slavery (Landman and Silverman 2019; Le Baron 2021). The UK's Modern Slavery Act (MSA) also targets large companies, but is notable for requesting that businesses report details about the modern slavery risks identified within their operations and supply chains, along with what actions are being undertaken in response, but stops short of formally enforcing compliance (Le Baron 2021). Instead, the MSA arguably represents an attempt to involve consumers, workers, and other stakeholders in the process of monitoring and pressuring company action, reflecting a view that the wider participation of society is essential to ensuring antislavery measures are undertaken (Carrington, Chatzidakis, and Shaw 2021).

Complicating these assumptions is the concern that modern slavery is widely known to exhibit a number of distal features and properties that could influence its appraisal and what resulting action by individuals may follow (Andersson et al. 2019; Carrington, Chatzidakis, and Shaw 2021; Simpson et al. 2021). In particular, people's perception of the proximal distance of many modern slavery cases has been highlighted as one reason that businesses and consumers commonly display apathy towards the problem because “far away” threats often have few direct impacts and elicit little immediate concern to individuals (Carrington et al. 2021). Work by Carrington et al. (2021) also finds proximal considerations to be variously woven into techniques of “neutralization” and “legitimization,” which help individuals ameliorate the intensity of ethical dilemmas and to justify not taking preventative action. For instance, victims of modern slavery may attract little sympathy amongst consumers if they are perceived to reside in places where cultural and political practices are considered to be radically different (Carrington et al. 2021). In other respects, adults who fall victim to modern slavery are also to be deemed by some to deserve less sympathy compared to children, following assumptions that they have more agency to escape harsh labor conditions and may simply choose not to do so (Carrington et al. 2021). To some, modern slavery may also be argued to represent an inevitable consequence of capitalism and therefore warrant little response following the expectation that “exploitative labor” practices are integral to the production of cheap goods and services around the world (Banerjee 2021).

In the workplace, such perceptions and justifications are also likely fueled, in part, by the opacity of economic relations and processes due to issues such as the complexity and depth of global supply chains, which can contain many tiers allowing modern slavery to thrive while being kept from view (Benstead et al. 2021; Trautrims et al. 2020). Nevertheless, even when incidences of modern slavery are known to be “localized,” its practice can remain “hidden in plain sight” and hard to identify despite suspicions of illicit activity (Bales 1999). A further potential difficulty is the possibility of the “bystander effect,” whereby people fail to act due to modern slavery being perceived as a “low emergency threat” amidst high levels of ambiguity surrounding its practice, whose responsibility it is to intervene, and what incentives there may be to do so (Stevenson 2022). For these reasons, modern slavery continues to be considered an “unseen problem” that whilst undesirable remains both widespread and hard to stop.

Yet, despite these distal constraints, as with other widespread problems, individuals may still play a contributory, if not determining, role in identifying and mitigating the risk of modern slavery (Carrington et al. 2021; Stevenson 2022). Current research suggests that, as consumers, individuals can put pressure on the producers of goods and services through such means as their purchasing decisions, and may look for signs of a company's ethical credentials when doing so (Stringer et al. 2022). Meanwhile, at work, other studies similarly indicate that modern slavery can elicit ethical worries and prompt individuals into action depending on the features they attend to (Carrington et al. 2021; Simpson et al. 2021). Moreover, in the absence of wider structural reform, the actions of individuals may have the biggest impact on what organizational efforts are taken to mitigate the risk of modern slavery (New 2015). Further research is therefore needed to increase understanding of the role of micro‐level factors in shaping the extent to which modern slavery is identified by individuals as a risk of concern that warrants action to help mitigate its practice.

### 2.3 Conceptual Model and Hypothesis Development

**FIGURE 1:**
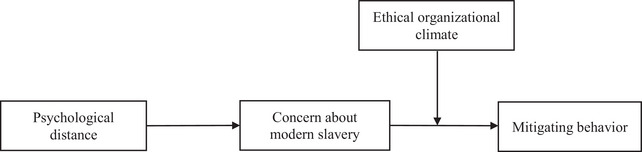
Conceptual model.

In this study, we develop and empirically test a conceptual model specifying how the psychological distance of modern slavery risk is related to individual levels of concern and intentions to engage in mitigating behavior, as well as the role of ethical climate in the workplace in shaping these associations (see the conceptual model presented in Figure 1). Next, we elaborate our theoretical rationale and hypotheses for testing this proposed model.

#### 2.3.1 The Psychological Distance of Modern Slavery

In line with previous research, we consider that several key features of modern slavery are distal in character and therefore that psychological distance will play an important role in how people appraise and respond to this risk. Modern slavery is commonly a hidden practice that is hard to recognize and detect due to aspects such as a lack of transparency regarding complex, cross‐border supply chains (Kougkoulos et al. 2021). And while legislation, such as the UK's MSA, might require large firms to assess their modern slavery risks, this often does not always lead to robust audits or firm‐wide awareness (Stevenson 2022). Subsequently, employees may be left largely unaware or unsure of how modern slavery risks affect their organizations. These considerations are also reflected in research examining the related problem of “human trafficking,” which found that people who do not easily identify or recognize this crime generally do not advocate for its victims (Sigmon 2008). Therefore, for many individuals, the problem of modern slavery is broadly beset by numerous factors such as low awareness, ambiguity about its practice, little consensus regarding its mitigation, and few direct lines of responsibility for addressing the problem (Loy and Spence 2020). We propose that the incidence of psychological distance will accordingly exacerbate inaction towards this problem.

Greater psychological distance is negatively related to intention to engage in mitigating behaviors.

#### 2.3.2 The Role of Concern as a Mediator Between Psychological Distance and Engagement With Mitigating Behaviors

Concern is generally considered an important antecedent to actions taken by individuals to reduce or eliminate a risk; if an individual is not concerned about an issue, they are much less likely to attend to it, speak up, or do anything else about it (Heaphy, Lilius, and Feldman 2022; Wardman 2006). This is demonstrated across a range of cases, where studies show, for example, that heightened concern about threats—ranging from climate change to COVID‐19—plays a key role in shaping the propensity of individuals and organizations to undertake mitigating actions (Cakir et al. 2023; Spence et al. 2021). Subsequently, various communication campaigns and education programs have attempted to raise concern to help address a wide spectrum of risk‐related issues (Dahlmann and Roehrich 2019).

Studies of psychological distance have in turn indicated that, when psychological distance is high, there tends to be a low level of concern, whereas conversely if psychological distance is low this is likely to lead to higher levels of concern amongst individuals (Spence et al. 2012). The tendency of people to exhibit lower levels of concern is most evident when they consider its impacts to affect only “other” people or nations (Trope et al. 2007). These connections are also illustrated by research findings that public concern about human trafficking is shaped by perceptions of the proximity and prevalence of the crime, as well as factors such as the portrayal of the difficulties faced by immigrants in the news (Bishop et al. 2013). In recent times, modern slavery has constituted an uncharted territory for many businesses, which have not considered it as a type of business risk and so not often raised it as a concern for workers (Simpson et al. 2021; Taylor and Shih 2019). National campaigns have, however, attempted to raise wider concerns about modern slavery risk, and the introduction of legislation has been intended to put pressure on companies to adopt new protocols to act on the issue in the workplace (Benstead et al. 2021). Therefore, we propose that the psychological distance of modern slavery amongst workers will also be important to levels of concern about this risk, which in turn will help to shape behavioral intentions to act on the issue.

Greater psychological distance is negatively related to levels of concern about modern slavery.

Concern about modern slavery is positively related to intention to engage in mitigating behaviors.

The relationship between psychological distance and intention to engage in mitigating behaviors is mediated by concern about modern slavery.

#### 2.3.3 Moderating Role of Ethical Organizational Climate

Human rights abuses surrounding the practice of modern slavery risk often prompt moral considerations (Carrington et al. 2021; Stringer et al. 2022). Within organizations, ethical climate can be defined as “the shared perception of what is correct behavior, and how ethical situations should be handled” (Victor and Cullen 1987, p. 51). Research suggests that ethical climate within an organization emerges through group interaction and the values conveyed by top management, and so helps to shape moral awareness, attitudes, and behaviors by giving employees a sense of “how things are done” (Lu and Lin 2014; VanSandt et al. 2006; Wang and Hsieh 2013). Likewise, working in a strong ethical organizational climate in which team members share moral standards of fairness, trust, and support can encourage empathetic concern (David et al. 2021; Victor and Cullen 1987). As modern slavery is considered an ethical issue that extends beyond purely legal consequences (Dahan and Gittens 2010; Trautrims et al. 2020), this makes ethical organizational climate a potentially important boundary condition for engagement in mitigating behaviors.

Moreover, whilst many firms have historically strengthened efforts to integrate social sustainability goals into their work practices, issues such as modern slavery can remain largely overlooked (Von Geibler et al. 2006). Subsequently, many companies do not have well‐established strategic goals and formal procedures to address modern slavery risks (Stevenson 2022). This tendency makes ethical organizational climate important as employees will rely on interpersonal social cues as a guide to concern and action about modern slavery risk. This might occur, on the one hand, where there are established working practices and norms that prioritize operational efficiency and profit maximization over all other considerations, which then diverts employees from raising concerns and pursuing options that are fairer, more sustainable, and better for human well‐being and the environment (David et al. 2021). In such cases, employees may keep silent knowing they will not be held accountable for engaging in (un)ethical actions (Heaphy, Lilius, and Feldman 2022; Wang and Hsieh 2013). On the other hand, employees in organizations that have a strong ethical climate are more likely to reflect on the moral implications of activities, such as how modern slavery might affect business operations and supply chains, and incorporate this into the workplace (Kuenzi et al. 2020).

The relationship between concern about modern slavery and intention to engage in mitigating behavior is stronger when ethical organizational climate is stronger.

Studies indicate that the strength of links between psychological distance and mitigating behaviors can depend on “boundary conditions” associated with the social, political, or organizational context in which individual action is undertaken (e.g., Chu and Yang 2020). For example, one factor that weakens the impact of psychological distance on mitigation behavior is feeling overwhelmed by the magnitude of a problem, such that an individual considers efforts to help solve grand challenges as pointless (Brügger et al. 2015; Feinberg and Willer 2011). There is also the further related issue that individuals may engage in techniques of legitimization and justification, which lessen the intensity of moral concerns and lead to inaction (Carrington et al. 2021)

Nevertheless, ethical organizational climate can help to provide well‐established sociocultural norms and clear organizational cues to steer judgments and decisions in directions that follow core business values even in cases where new problems seem complex, multifaceted, or morally ambiguous (Cakir et al. 2023; David et al. 2021; Kuenzi et al. 2020). By providing cues about the appropriateness of morally guided conduct in the workplace and supporting a sense that individuals can meaningfully contribute to antislavery measures, a strong ethical organizational climate can accordingly help to offset barriers to action otherwise faced by employees. We therefore further predict that ethical organizational climate will influence the mediated relationship between psychological distance and mitigating behavior.

The strength of the mediated relationship between psychological distance and mitigating behavior (through concern about modern slavery) is moderated by ethical organizational climate; the indirect effect of psychological distance on mitigating behavior will be stronger when ethical organizational climate is high.

## 3 Method

### 3.1 Sample and Procedures

We recruited participants via a third‐party provider (i.e., Qualtrics), which invited participants from the United Kingdom to our study via its platform in return for compensation. We asked Qualtrics to recruit participants only if they are working full‐time and having a minimum of one‐year work experience in their current organizations in order to ensure some familiarity with organizations’ ethical values and standards. Of the 5304 individuals who wanted to take part in our study, 3209 were removed as they did not satisfy the above eligibility criteria. Another 1262 were removed due to failing attention checks, and finally 322 failed to complete the study on time, which resulted in a final sample size of 511. Participants in the final sample had an average age of 43.93 (*SD* = 10.70), holding an average work experience of 23.13 years (*SD* = 11.47), and 54% were female. The average tenure of participants in the final sample was 9.31 years (*SD* = 8.44), which is in line with previous empirical studies using the ethical climate work climate construct (e.g., Mayer et al. 2010; Kuenzi et al. 2020). Table 1 provides further details about participants.

**TABLE 1:** Characteristics of participants.

Characteristics of participants		Number	Frequency
Education level	No formal qualifications	3	0.6
	High school diploma	152	13.1
	Professional qualification	56	11
	Undergraduate degree	196	38.4
	Postgraduate degree	104	16.8
Managerial level	Nonmanagerial role	273	53.4
	Manager	105	20.6
	Middle manager	88	17.2
	Senior manager	30	5.9
	Executive	15	2.9
Industry sector	Automotive and related equipment	23	4.5
	Construction and related products	43	8.4
	Education	102	20
	Electronics and IT	67	13.1
	Financial and insurance activities	48	9.4
	Food and beverages	19	3.7
	Logistics	24	4.7
	Medicine, chemical, and pharma	62	12.1
	Textile	69	13.5
	Other	54	10.6
Department	Finance	63	12.3
	General management	123	24.1
	Human resources	45	8.8
	Production	110	21.5
	Research and development	62	12.1
	Sales/marketing	69	13.5
	Supply chain/procurement	39	7.6

We did several tests to examine systematic biases in our final sample. First, we compared the responses of early and late participants, which showed there is no significant difference (*p* > 0.10). Then, we compared our final sample with participants who dropped out before the end of the survey in terms of their age and tenure in their organizations. Again, we found no statistically significant differences between these groups (*p* > 0.10). We concluded that the final sample was not influenced by a systematic bias.

### 3.2 Materials

In order to minimize conceptual ambiguity, participants were provided with a definition of modern slavery alongside the questions. We adopted the definition provided by the Independent Anti‐Slavery Commissioner of the United Kingdom:

Modern slavery is an umbrella term encompassing, but not limited to: slavery, servitude, forced or compulsory labor and human trafficking. Its victims are unable to leave their situation of exploitation, often controlled by threats, punishment, violence, coercion, grooming and deception.

#### 3.2.1 Psychological Distance

We developed a new 17‐item instrument to measure psychological distance to modern slavery, adapted from items provided by Jones et al. (2017), Spence et al. (2012), and Wang et al. (2019). One sample item is “I think about countries far away when thinking of modern slavery.” Possible response options ranged from *strongly disagree* (= 1) to *strongly agree* (= 5). Details of the instrument development process are reported in the Appendix.

#### 3.2.2 Concern About Modern Slavery

We measured concern about modern slavery using seven items adapted from previous studies (Jones et al. 2017; Spence et al. 2012; Wang et al. 2019). Possible response options ranged from *very concerned* (= 1) to *not at all concerned* (= 4). One sample item is “How concerned, if at all, are you about modern slavery, sometimes referred to as forced labor?”

#### 3.2.3 Ethical Organizational Climate

We used the Kuenzi et al. (2020) 12‐item measure to assess an organization's ethical climate. Possible response options ranged from *strongly disagree* (= 1) to *strongly agree* (= 5) to items such as “Employees receive positive feedback for making ethical decisions.”

#### 3.2.4 Mitigating Behaviors

We measured participants’ intentions to adopt mitigating behaviors with seven items. Possible response options ranged from *very unlikely* (= 1) to *very likely* (= 4) for items such as “I intend to discuss modern slavery risk with colleagues.”

#### 3.2.5 Control Variables

Consistent with previous research (Jones et al. 2017; Linde 2020), we incorporated age and gender into our analyses as control variables.

### 3.3 Reliability and Validity

We used Cronbach's alpha to assess the internal reliability of the measures used in our study. As can be seen in Table 2, all measures had Cronbach's alphas well above the recommended threshold of 0.70, indicating good internal reliability for all measures (Nunnally 1978).

**TABLE 2:** Descriptive statistics and correlations.

	1	2	3	4	5	6	Mean	*SD*
1. Age	_						43.93	10.71
2. Gender	−0.18^**^	_					1.54	0.50
3. Psychological distance	−0.01	−0.12^**^	**0.92**				2.64	0.69
4. Concern about modern slavery	−0.08	0.14^**^	−0.37^**^	**0.88**			2.98	0.61
5. Ethical organizational climate	−0.17^**^	0.07	0.06	0.19^**^	**0.95**		3.46	0.95
6. Mitigating behavior	−–0.18^**^	0.07	−0.15^**^	0.55^**^	0.37^**^	**0.91**	2.27	0.70

*Note: N* = 511. Cronbach's alpha coefficients are presented in bold in the leading diagonal. *p < 0.05, **p < 0.01.

We then conducted confirmatory factor analysis to assess the discriminant and convergent validity of our measures. The full measurement model with all latent variables yielded acceptable model fit (χ2/*df* = 2.61; comparative fit index [CFI] = 0.92; Tucker–Lewis index [TLI] = 0.91, root mean square error of approximation [RMSEA] = 0.06). We compared this baseline model against theoretically plausible alternative nested models. In the best performing nested model, we loaded moderating (ethical organizational climate) and mediating (concern about modern slavery) variable items onto one factor. The results showed that the measurement model fit data better than the best performing nested model (χ2/*df* = 4.40; CFI = 0.83; TLI = 0.82, RMSEA = 0.08), which provided evidence for discriminant validity. We assessed the convergent validity of our measures by computing average variance extracted (AVE). All measures have AVEs above the threshold of 0.5 (Fornell and Larcker 1981), suggesting sufficient convergent validity.

### 3.4 Common Method Bias

We followed several data collection procedures to minimize common method bias risks. First, participants of the study ascertained that their responses would be presented anonymously and in an aggregated format. Second, we separated independent and dependent variables and randomized items within constructs. Finally, we employed some key selection criteria to make sure participants had the required knowledge for answering our questions and that they completed the study with due attention.

After collecting the data, we undertook some further checks to assess common method bias risk in the final dataset. First, we ran Harman's one‐factor test to assess whether a common factor would explain more than 50% of the variance. The results show that a single factor did not account for the majority of variance, suggesting that the common method bias is not a serious concern. Second, we used a marker variable test to examine common method variance as responses to independent and dependent variables are obtained from the same source (Lindell and Whitney 2001). We chose a three‐item ethical ambiguity in the workplace measure by Johlke and Duhan (2000) (Cronbach's alpha = 0.88), which is theoretically unrelated to at least one of the variables in the study. We selected the lowest correlation between ethical ambiguity in the workplace and study variables (*p* = 0.04 between ethical ambiguity and psychological distance to modern slavery). The significance and directions of marker variable‐adjusted correlations were not different from the zero‐order correlations, indicating that common method bias is not a serious concern.

We also assessed the multicollinearity among variables. As shown in Table 2, none of the correlations among measures were above the cutoff value of 0.70 (Tabachnick and Fidell 2013). We also compute variance inflation (VIF) scores. The highest VIF score was 1.2, which is well below the threshold of 2.5, indicating an absence of multicollinearity.

## 4 Results

**FIGURE 2:**
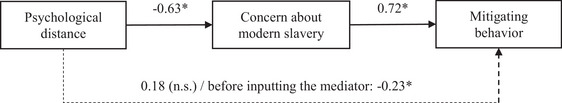
Results of structural path model. *Note*: *p < 0.01.

Our findings indicate a “moderate” to “strong” level of psychological distance towards modern slavery on average, along with a good distribution of ratings, reported by participants in our study (*M* = 2.64, *SD* = 0.69). To test our hypotheses, we first specified a structural equation model using SPSS AMOS 29.0, which included all direct and indirect effects in our conceptual model (H1, H2a, H2b, H3). The specified model presented in Figure 2 demonstrated a good fit with our observed data (χ2/*df* = 3.13; CFI = 0.91; TLI = 0.90, RMSEA = 0.06). Among the control variables, age (*β =* −0.01, *p <* 0.01) was significantly linked to intention to engage in mitigating behavior. Among the main effect variables, psychological distance had a negative impact on concern about modern slavery (*β =* −0.63, *p <* 0.01), thus supporting H2a. Furthermore, concern about modern slavery was positively related to intention to engage in mitigating behavior, supporting H2b.

Psychological distance (*β =* −0.23, *p <* 0.01) had a significant direct impact on intention to engage in mitigating behavior when the mediator (i.e., concern about modern slavery) is not included in the model, thus supporting Hypothesis 1. However, the direct effect of psychological distance becomes insignificant (*p >* 0.05) when the mediator (i.e., concern about modern slavery) is added to the model, providing support for our Hypothesis 3, which posits that concern about modern slavery mediates the relationship between psychological distance and intention to engage in mitigating behavior. We further analyzed the mediating effect of concern about modern slavery by generating a bootstrap bias‐corrected interval based on 5000 samples (Hayes 2013). The 95% bootstrap confidence interval (CI) did not contain zero (CI = [−0.27, −0.14], *p* < 0.01), which provided further support for Hypothesis 3.

**FIGURE 3:**
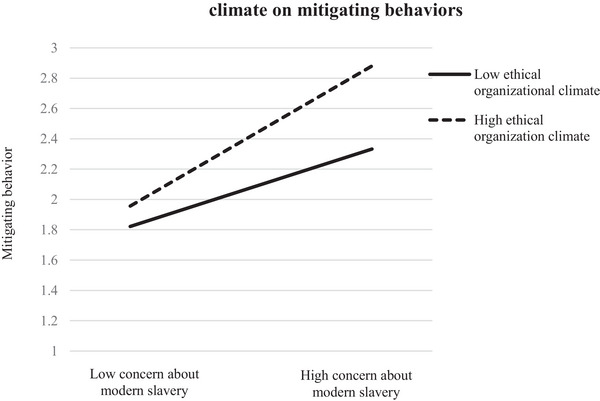
Interaction of concern about modern slavery with ethical organizational.

Hypothesis 4 proposes that ethical organizational climate moderates the relationship between concern about modern slavery and intention to engage in mitigating behaviors. We used PROCESS Model 1 macro with 5000 bias‐corrected bootstrapped samples to test the moderating impact of ethical climate. Results indicate that the interaction term (psychological distance × ethical organizational climate) has a significant impact on intention to engage in mitigating behaviors (*β =* 0.18, *p <* 0.001), which provides support for Hypothesis 4. To provide insight into this interaction pattern, we plotted the relationship between these variables. Accordingly, Figure 3 presents the relationship between concern about modern slavery and intention to engage in mitigating behaviors at high and low levels of ethical climate by values one standard deviation above and below the mean (Aiken and West 1991). This shows that people's concern about modern slavery has greater influence on intention to engage in mitigating behaviors when ethical climate is high.

Finally, Hypothesis 5 predicts a moderated mediation in which ethical climate moderates the indirect effect of psychological distance on intention to engage in mitigating behavior via concern about modern slavery. To do so, we use the PROCESS Model 14 macro and calculate the index of moderated mediation (Hayes 2013). The index was significant (*p <* 0.01), and the CI did not include zero (CI = [−0.10, −0.02]). Table 3 shows an indirect effect of psychological distance on intention to engage in mitigating behavior that is significant at high and low levels of ethical climate, thus supporting Hypothesis 5.

**TABLE 3:** Bootstrapped results for the conditional indirect effects.

			95% bias‐corrected confidence intervals	
Conditional indirect effect via concern about modern slavery	Coefficient	SE	LL	UL
−1 standard deviation	−0.13	0.03	−0.2	−0.08
+1 standard deviation	−0.24	0.04	−0.32	−0.17
	Index	SE	LL	UL
Index of moderated mediation	−0.06	0.02	−0.10	−0.02

*Note*: 5000 bootstrapping samples. Abbreviations: LL, lower limit; SE, standard error; UL, upper limit.

## 5 Discussion

Our findings make some novel contributions to current research and policy understandings of people's appraisals of modern slavery risk in the workplace. First, we show that psychological distance is prevalent amongst our sample of UK workers. Second, higher levels of psychological distance are shown to be associated with lower levels of individual concern about modern slavery risk as well as a lower intention to undertake mitigating actions. Third, these relations are further found to be moderated by ethical climate, with a stronger ethical climate intensifying the positive impact of risk concerns on the greater intention by individuals to take mitigating actions against modern slavery. This study therefore underlines the key potential for targeted micro‐level interventions in the workplace to reduce psychological distance by such means as raising awareness and understanding of the proximal incidence and impacts of modern slavery. Our findings also highlight the value of promoting a strong ethical climate within organizations to reduce psychological distance and enhance the use of antislavery action by workers. These considerations may be particularly important, both theoretically and practically, in the absence of a wider government mandate for organizations to address modern slavery risk, as well as for enhancing the effectiveness of new antislavery initiatives that are introduced.

### 5.1 Theoretical Contributions

Building on previous studies, we extend research on construal‐level theory into the new context of modern slavery. The findings of prior qualitative research indicate that psychological distance can affect the nature of risk perceptions in terms of the focus given to different salient features of modern slavery risk and what to do about it (Simpson et al. 2021; Stringer et al. 2022). This study empirically confirms the importance of psychological distance to micro‐level understandings and appraisals of modern slavery by showing that it is widespread amongst our sample of workers across different industries. We further demonstrate that psychological distance affects levels of concern about this risk and that this influences behavioral intentions to act to mitigate this risk as the level of concern rises.

Next, we extend the key considerations of recent work on construal level theory to include ethical climate as a conceptually relevant boundary condition. Particularly, we show that ethical climate moderates the impact of psychological distance on individuals’ behavioral intentions to mitigate modern slavery within an organizational context. This finding underscores the importance of moral norms and outlooks to the construal of modern slavery risk in the workplace. This may be especially important given prior research, which indicates that individuals often find ways to justify inaction and reduce the intensity of moral concern (Carrington et al. 2021). Following which, the socially supportive features of a strong ethical climate may in turn be important to helping offset those tendencies to downplay the moral aspects of distal risks. For example, problems or threats with distal qualities may require a strong ethical climate to support employee communications around otherwise difficult or unwelcome issues of concern (Cakir et al. 2023). Taken together, these theoretical contributions help to contribute towards a fuller picture of the micro‐level components and processes related to the construal of distal features of modern slavery risk and how this is important to appraisals and behaviors.

We also extend the literature on modern slavery in several ways. To the best of our knowledge, our study offers one of the first quantitative examinations of individual perceptions of modern slavery and their behavioral implications. While most quantitative studies have concentrated on national‐level estimates of modern slavery prevalence, little is known about how individuals perceive and respond to modern slavery risk in the workplace (see Caruana et al. 2021 for a review). Furthermore, there are no validated tools available to assess individual perceptions related to modern slavery. Addressing these gaps, we develop and validate a new measurement instrument to assess people's psychological distance to modern slavery, and we test its explanatory power in predicting mitigating behaviors in the workplace. Our findings highlight the potential of psychological microfoundations in shaping antislavery actions at the individual level within organizations. We hope our findings, along with the new measure, will encourage greater incorporation of micro‐level perspectives into modern slavery research.

### 5.2 Practical Implications

In practical terms, our study findings have relevant implications for both policymakers and managers. First, we provide clear evidence that lower psychological distance is associated with greater intention to engage in mitigating behavior. This finding therefore supports the use of interventions designed to reduce psychological distance and promote mitigating behaviors against modern slavery in the workplace. A number of organizational strategies might be employed in these regards, including informational campaigns and training to draw attention to the risk and to raise awareness to redress proximal considerations of modern slavery as an uncertain and far away threat that only happens to other people. To support training and evaluation, organizations could also administer our comprehensive measure of psychological distance to modern slavery as a diagnostic tool to evaluate people's perceptions of modern slavery and how these might change following interventions. For instance, while organizations might focus on the incidents of modern slavery happening in the United Kingdom in their communication to reduce geographical dimension of psychological distance, they can stress that modern slavery does not only happen in emerging or low‐income economies but also in advanced economies to target social dimension of psychological distance. Similarly, for policymakers, a better understanding of psychological distance may provide key insights about where to target communication strategies and what focus is most required.

A second practical implication concerns the promotion of ethical climate as a means to enhance concern and mitigate behavior. Organizations can foster ethical climate in several ways. For instance, managers can affirm the importance of maintaining moral standards in general communications regarding expectations of moral conduct through such means as the inclusion of moral considerations in mission statements and the use of a comprehensive code of ethics. Recruitment and training can also support the understanding of ethical issues and dilemmas by employees. Managers can also make sure performance appraisals reward employees’ engagement with ethical behavior.

### 5.3 Research Limitations and Future Directions

Whilst, to our knowledge, this study provides the first quantitative examination of psychological distance and perceptions of modern slavery risk amongst workers across industry, our inquiry was undertaken with employees in UK businesses. The United Kingdom has a particular history regarding recent efforts to recognize, assess, and mitigate the risk of modern slavery, and while some of the efforts have prompted criticism, the United Kingdom is still considered a “world leader” on this issue through the use of antislavery initiatives that have notably included the novel introduction of a MSA (2015), which has specifically targeted industry. This national context may have contributed to the general level of psychological distance observed within our sample. We suggest that it would be worthwhile to conduct further comparative studies across different regional, national, and cultural contexts to examine the wider replicability of our findings, as well as the broader applicability of our research instruments.

Our findings align with previous inquiry into people's construal of distal threats and sustainability issues such as climate change (Kirshner and Moritz 2023; Spence et al. 2012). We suggest, accordingly, that there may be further value to incorporating other insights and understandings from this research literature. For example, one recent line of inquiry has concerned the multidimensional components of psychological distance (e.g., location, uncertainty) and how differences in the level of construal of each dimension can variably impact people's conceptualization and perception of sustainability‐related phenomena (e.g., climate change) and their subsequent behaviors (Loy and Spence 2020). We suggest that our findings provide good grounds for future modern slavery studies to extend inquiry along these lines by incorporating more elaborate sociocultural models and conceptual research designs to test the mutual correspondence and individual influence of respective components of psychological distance and their impact on risk responses.

In our study, the research focus concerned individual appraisals of modern slavery risk in the workplace, but the additional consideration of ethical climate could also be meaningfully applied to other social and organizational domains For instance, beyond the workplace, consumption choices might be influenced by perceptions of the ethical values espoused by businesses particularly where consumers strongly identify with company branding (Stringer et al. 2022); and vice versa, companies may adopt more “sustainable” procurement and production practices where these values align with the preferences of investors alongside those of target consumers (New 2015). More generally, individuals typically undertake many other activities, whether at home, in their neighborhood, or across their wider community, that involve group identification and interaction, such as when participating in sports and forms of entertainment, or religious worship, for example. Such activities may also extend to interactions within online communities via social media. These sociocultural contexts will also have their own “ethical climates” and so provide further avenues to test the broader applicability of the conceptual model employed in this study.

Additionally, future research studies could also examine what other mediators might influence the link between psychological distance and intentions to engage in mitigating behaviors across different contexts of modern slavery. For example, research by Chu and Yang (2020) indicates that at closer psychological distance, intentions to engage in mitigating behavior regarding climate change are shaped by perceived “efficacy” and so recommend that message frames should aim to boost efficacy in this context; whereas for risks construed at greater distance and levels of abstraction, perceived risk plays a stronger role, therefore messaging should adopt a risk‐based frame highlighting the disastrous consequences to encourage action. For modern slavery, research has indicated that considerations of “low efficacy” play an important part in reducing the intensity of moral concerns and supporting justification for inaction against (Carrington et al. 2021). However, to date, these considerations have not been extended to testing the association of efficacy with psychological distance and their combined impacts on behavior, which seem ripe for exploration. Scholars might also benefit from recent research on climate action, which would provide further plausible mediators (e.g., construal level, legacy motive) and moderators (e.g., cultural background and social norms) applicable to the modern slavery context (see Kim 2023, 2024)

Organizational communication with workers and wider stakeholders is also likely to vary widely between companies, and these possible differences offer a further avenue for future inquiry. For instance, some organizations may undertake extensive efforts to share information about the company values which shape ethical climate, whereas others may do so only tacitly, and differences in the moral content of that information may also be important to individual perceptions (Cakir et al. 2023). Similarly, the distal framing and “modality” of messages used in communications about modern slavery may also be another important consideration for interventions. For example, research on distal focus by Lui and Yang (2023) shows that the type of “narrative persuasion” employed in environmental messaging can shape its effectiveness, with the “social closeness” of target objects depicted increasing message persuasiveness, especially when there is closer identification by recipients. Recent interest in “survivor narratives” within modern slavery studies has also begun to elaborate how different aspects of its practice (e.g., trafficking, physical isolation, language barriers, and psychological coercion) can figure in understandings of the causes and impacts of modern slavery in society (Le Baron 2021). Recent work by Valenzuela et al. (2023) has likewise pointed to the benefits of foregrounding the vulnerabilities and subjectivities of those in precarious work. However, research has yet to examine how messaging that incorporates narrative accounts based on such personal subjectivities might accordingly help to bring a more humanized dimension to understandings of modern slavery, thereby helping to reduce psychological distance, as well as increase actions taken against this risk.

Finally, our results are based on cross‐sectional data, which limits our capacity to draw causal inferences regarding associations proposed in the conceptual model. Future studies can employ more rigorous research designs (e.g., that utilize an experimental or longitudinal approach) to provide evidence for causal relationships and to test the impact of interventions.

## 6 Conclusion

Our study addresses an important gap in modern slavery research and complements previous studies into psychological distance and the construal of distal risks more generally. Whilst addressing psychological distance may not provide a “magic bullet” for mitigating the problem of modern slavery, empirical support for our proposed model and analysis of these relations underscores the importance of understanding and targeting the microfoundations of people's workplace understandings and behavior regarding this risk. By extension, we propose that policy interventions may benefit from further research into this promising line of inquiry as studies expand understandings of the distal character and contextual features of the psychological distance of modern slavery risk, as well as different mediating influences that achieve prominence across different circumstances and contexts. The resulting insights generated could in turn help to enhance the effectiveness of interventions targeting the reduction of psychological distance, prompt further vigilance, help reduce barriers to action, and ultimately support mitigating behavior undertaken in response to this distal risk.

## 1

In the following sections, we describe our procedure for the construct validation of psychological distance to modern slavery scale. As there was no previous measure for the psychological distance of modern slavery, we had to develop a new measure. Construal level theory proposes that psychological distance has four dimensions, namely geographic, temporal, social, and uncertainty (Trope and Liberman 2010). Consistent with prior research, our new measurement instrument was grounded in this theory, with items designed to capture these four dimensions of psychological distance.

As a first step in measurement development, we created a pool of items. In addition to creating new items, many of the items were adapted from previous studies examining the psychological distance of climate change risk (e.g., Jones et al. 2017; Spence et al. 2012; Wang et al. 2019). The main rationale behind incorporating insights from the climate change literature on measuring the psychological distance concerned that this is one of the most mature research literatures on this topic, and both modern slavery and climate change are sustainability risks that feature as part of the UN's Sustainable Development Goals agenda.

In the next step, we submitted the final pool of 31 items to an exploratory factor analysis to assess different dimensions of the psychological distance of modern slavery and achieve a parsimonious and reliable instrument. We used direct‐oblimin rotation as we assumed that different dimensions of psychological distance would be correlated. Items that loaded greater than 0.40 on a unique factor were retained to define the resulting factors (cf. Hinkin 1998; Tabachnick and Fidell 2013). Cattell's scree test was used to identify the number of factors to retain.

Table A1 presents the results of the exploratory factor analysis, which yielded a 17‐item, four‐factor solution, explaining 74.74% of the variance. All factors exhibit high internal reliabilities, with Cronbach's alphas well above the recommended threshold of 0.70 (Nunnally 1978). Items reflecting the four theoretical dimensions of psychological distance loaded uniquely on a particular factor.

**TABLE A1:** Pattern matrix pertaining to exploratory factor analysis.

	F1	F2	F3	F4
*F1: Geographical *				
1. I think about countries far away when thinking of modern slavery.	**0.88**	−0.01	−0.01	−0.05
2. I feel geographically far from the effects of modern slavery.	**0.85**	0.01	0.03	−0.04
3. The worst effects of modern slavery will be felt by countries far from where I live.	**0.81**	0.03	0.01	−0.04
4. Modern slavery will mostly affect areas that are far away from here.	**0.77**	−0.04	0.00	0.07
5. My local area is largely immune to modern slavery.	**0.69**	0.08	0.01	0.06
*F2: Temporal*				
6. Modern slavery is harming people right now.^a^	−0.03	**0.99**	−0.02	−0.06
7. Modern slavery is an immediate threat affecting people right now.^a^	−0.01	**0.94**	−0.01	0.02
8. The recent impacts of modern slavery mean we must tackle the issue now.^a^	−0.02	**0.86**	0.09	0.02
9. If modern slavery is to happen, it will happen in the remote future.	0.17	**0.61**	−0.06	0.11
*F3: Social*				
10. I don't see myself as someone who will be affected by modern slavery.	0.00	−0.01	**0.83**	0.04
11. Modern slavery is likely to have a big impact on people like me.^a^	−0.06	0.04	**0.82**	−0.07
12. I don't think modern slavery will significantly impact people I know.	0.09	−0.03	**0.74**	0.05
13. The biggest impacts of modern slavery will be felt by people other than people like me.	0.04	0.00	**0.73**	−0.01
*F4: Uncertainty*				
14. The seriousness of modern slavery is exaggerated.	0.02	0.00	−0.02	**0.91**
15. I am uncertain that modern slavery is really happening.	0.00	0.01	−0.03	**0.80**
16. Most researchers agree that there are victims of modern slavery.^a^	−0.14	0.13	0.02	**0.78**
17. It is uncertain what the effects of modern slavery will be.	0.20	−0.11	0.06	**0.54**
Eigenvalues	7.39	2.59	1.48	1.25
Total variance explained by each factor	43.45	15.25	8.69	7.34
Cumulative variance explained by the factors	43.45	58.71	67.40	74.74

*Note: N* = 511. Bold is used to highlight the loading between an item and its respective scale/factor. ^a^Reverse scored.

After achieving a parsimonious structure, we evaluated the model fit of the proposed four‐factor model reported in exploratory factor analysis (EFA) against a series of alternative nested models using confirmatory factor analysis (CFA). Accordingly, we first compared the proposed four‐factor model with two three‐factor models in which the items of two psychological distance dimensions were specified to load into a single factor (Models 2–4). Then, we compared it with an alternative one‐factor model in which all items were specified to load into a one common factor (Model 5). The results presented in Table A2 show that the proposed model had a good fit with data (χ2/*df* = 3.41, CFI = 0.95, TLI = 0.94, RMSEA = 0.07). However, alternative nested models did not have adequate fit. Therefore, CFA results offer further support for the discriminant validity for the measures used in this study.

**TABLE A2:** Confirmatory tests of the discriminant validity of the measures.

Model name	χ2(*df*)	Δχ2(Δ*df*)	χ2/*df*	CFI	TLI	RMSEA
1: Proposed 4‐factor model	377.15 (113)		3.34	0.96	0.95	0.07
Alternative 3‐factor models						
2: Merging social and temporal dimensions	1365.15 (116)	988 (3)	11.77	0.79	0.76	0.14
3: Merging geographical and social dimensions	940.88 (116)	563.73 (3)	8.11	0.86	0.84	0.12
4: Merging temporal and uncertainty dimensions	983.24 (116)	606.09 (3)	8.48	0.86	0.83	0.12
5: Alternative 1‐factor model	2960.58 (119)	2,583.43 (6)	24.88	0.53	0.46	0.22

*Note*: *N* = 511. Abbreviations: CFI, comparative fit index; TLI, Tucker–Lewis index; RMSEA, root mean square error of approximation. **p* < 0.01.

There is no consensus on the dimensionality of psychological distance, as studies have used both unidimensional and multidimensional measures in empirical research (Spence et al. 2012; Wang et al. 2019). Accordingly, after establishing the first‐order factors, we also assessed the viability of a second‐order factor (i.e., four first‐order factors leading to a second‐order factor), which would represent people's overall psychological distance to modern slavery. We followed Johnson et al. 2011, 2012) three criteria to provide an empirically justified second‐order factor, specified as follows: (i) the second‐order model should exhibit good fit with the data, (ii) first‐order dimensions should have substantial loadings on the second‐order factor, and (iii) the second‐order factor has strong internal reliability.

The second‐order model incorporating four first‐order factors and a second‐order factor had a good fit with the data (χ2/*df* = 3.94; CFI = 0.94; TLI = 0.93; RMSEA = 0.07). Factor loadings for the second‐order factor were significant (*p* < 0.01) and substantial in size, with an average of 0.70. Finally, the composite latent variability of the second‐order factor was 0.86, suggesting that the second‐order psychological distance measure exhibits good internal reliability. Overall, the results lend strong support for the higher order, unidimensional scale incorporating the four first‐order dimensions.
